# Intolerance of uncertainty causally affects indecisiveness

**DOI:** 10.1111/bjc.12534

**Published:** 2025-03-11

**Authors:** Helmut Appel, Alexander L. Gerlach

**Affiliations:** ^1^ Institute of Clinical Psychology and Psychotherapy University of Cologne Cologne Germany

**Keywords:** decision making, indecisiveness, intolerance of uncertainty, transdiagnostic factors

## Abstract

**Objectives:**

Intolerance of uncertainty (IU) is characterized by a pervasive negative reaction to uncertainty. It is a transdiagnostic risk factor for various mental disorders. Since decisions often need to be made in the face of uncertainty, IU is associated with indecisiveness, a dispositional difficulty in making decisions. Indecisiveness is also linked to a range of mental disorders. While IU is seen as a causal factor in indecisiveness, experimental studies on this assumption are lacking.

**Methods:**

In this pre‐registered, adequately powered study (*N* = 301), IU was experimentally increased or decreased compared to a control group, and the effect on indecisiveness was observed. Indecisiveness was assessed in a situational context, focusing on two decisions that were personally relevant to participants.

**Results:**

The manipulation successfully affected IU. As predicted, increased IU led to more indecisiveness across both decisions compared to decreased IU. Exploratory analyses found that situational IU mediated the effect of the experimental manipulation on indecisiveness.

**Conclusions:**

The results are the first to demonstrate a causal effect of IU on indecisiveness, thus contributing to the explanation of indecisiveness and the role that uncertainty management plays in it. Moreover, they have implications for treating various mental disorders by highlighting the role of IU in the transdiagnostic phenomenon of indecisiveness.

**Practitioner points**

- This is the first study to show that intolerance of uncertainty—a pervasive negative reaction to uncertainty—has a causal effect on chronic decision‐making difficulties (i.e., indecisiveness).
- Both traits are associated transdiagnostically with symptoms of various mental disorders and are therefore therapeutically relevant.
- For patients presenting with indecisiveness, targeting intolerance of uncertainty may be an important component contributing to improvement.

## INTRODUCTION

Decision making—whether it involves a life‐ crossroads or a simple routine choice—profoundly influences our lives. The abundance of choices in modern life has not only heightened the importance of making sound decisions but also made the process more challenging (Vohs et al., 2008). In stark contrast, indecisiveness is a “trait‐like difficulty making decisions across time and situations” (Lauderdale & Oakes, 2021, p. 256). Although indecisiveness is a non‐pathological trait that some cultures even regard positively (Yates et al., 2010), elevated indecisiveness levels are associated with symptoms of several mental disorders, such as depression (e.g., Hallenbeck et al., 2022), obsessive‐compulsive disorder (e.g., Frost & Shows, 1993), or anxiety disorders (e.g., Rassin & Muris, 2005). Although indecisiveness is relatively under‐researched (Lauderdale & Oakes, 2021), the general relevance of decision making makes it an important trait, particularly when addressing disorders linked to increased indecisiveness. Supporting this perspective, high indecisiveness levels are associated with impairments in everyday life, such as procrastination on important tasks (Ferrari, 1994) or inadequate commitment to academic goals (Germeijs & De Boeck, 2002).

Although explanations for indecisiveness are likely multifaceted (van Randenborgh et al., 2010), Rassin  (2007) introduced a psychological model of indecisiveness specifying intolerance of uncertainty (IU, Freeston et al., 1994; Krohne, 1989) as a predisposing risk factor. IU is a personality trait defined as “an individual's dispositional incapacity to endure the aversive response triggered by the perceived absence of salient, key, or sufficient information, and sustained by the associated perception of uncertainty” (Carleton, 2016, p. 31). Like indecisiveness, high IU levels are found across mental disorders (McEvoy & Erceg‐Hurn, 2016), making IU a target for transdiagnostic psychotherapy treatments (e.g., Mofrad et al., 2020).

Crucially, IU seems to exert its influence—like increased negative emotions and decreased positive emotions in response to uncertainty (Morriss et al., 2023)—beyond actual threat or danger and can result from an aversion to uncertainty per se (Freeston et al., 2020). IU‐related reactions may therefore also occur in positive uncertain contexts, such as benign surprises (Pepperdine et al., 2018). That said, IU is also associated with altered appraisals of situations as being more uncertain or more threatening (Appel et al., 2024; Pepperdine et al., 2018). In other words, not only do high IU individuals dislike uncertainty more, but they also perceive more uncertainty and threat in a given situation. Given that most decisions involve some degree of uncertainty and often have unknown outcomes involving potential threat, it is not surprising that IU is seen as an interfering factor in decision‐making (Koerner et al., 2017; Rassin, 2007).

Accordingly, studies have demonstrated altered decision making as a function of IU (Carleton et al., 2016; Luhmann et al., 2011), as well as processes involved in decision making, like decreased decision certainty (Jensen et al., 2014), or more uncertainty‐reduction attempts (Jacoby et al., 2014). For example, Luhmann et al. (2011) had participants repeatedly choose between two options in a gambling task. Participants with higher IU levels more frequently chose an option with a lower value and a smaller chance of winning, merely to avoid a brief yet unpredictable (and therefore uncertain) delay before receiving the win/lose feedback. Looking at indecisiveness in particular, empirical findings show that it is robustly related to IU, both on a dispositional level (e.g., Koerner et al., 2017) and on a situational level in everyday decision making (Appel et al., 2024).

If IU is indeed a precursor to indecisiveness, this would be a strong argument for reducing IU as a way to help those looking to mitigate excessive indecisiveness. However, a causal effect of IU on indecisiveness has not been demonstrated so far (Rassin, 2007). In the present study, we therefore manipulated IU experimentally by randomly assigning participants to an Increase IU, Decrease IU, or control condition. In this way, we tested the causal effect of IU on indecisiveness. To ensure ecological validity, we worked with decisions personally relevant to the participants, thus using particularly strong methodology. We hypothesized that experimentally increasing IU would lead to greater indecisiveness compared to reducing IU (H1), and in comparison to a neutral control group where IU was not manipulated (H2). We also expected that reducing IU would lead to less indecisiveness compared to the neutral control group (H3).

## METHOD

The ethics board at the University of Cologne (reference no. HAHF0171) approved the study. Electronic supplemental materials (ESM) are provided on the OSF project website (osf.io/98u6r). Hypotheses and the analysis plan were pre‐registered (aspredicted.org/j3jy‐j77s.pdf). Any deviations from the pre‐registration were minor and are listed in Data S1.

### Participants

Considering the smallest effect size of interest (SESOI; Lakens et al., 2018), we aimed for a final sample of *N* = 300 participants in order to detect a small to medium size effect (approx. *d* = 0.3) in the pre‐registered main analysis (MANOVA, see Data Analysis) at 80% power. On the crowdsourcing platform Prolific.co™, 330 German native speakers gave informed consent and finished the study in exchange for ₤ 2.50 GBP. Following the preregistered exclusion criteria, we discarded data from participants who (a) failed the attention check (*n* = 2); (b) said we should not use their data (*n* = 1); (c) failed to remember essential parts of the instructions (*n* = 15); (d) did not produce at least one valid piece of advice (*n* = 1); (e) indicated they could “not at all” (1 on a scale from 1 to 5) think vividly about the decision (*n* = 6); and (f) did not describe a decision (*n* = 4). We had originally planned to apply criteria (e) and (f) only in analyses involving indecisiveness referring to participants' own decision, but for simplicity, we decided to completely exclude these cases. We tested the effect of these exclusions in sensitivity analyses (see Data Analysis). To determine criteria (d) and (f), two independent raters rated all advice and decision descriptions to ensure that the descriptions contained the requested content. Agreement was 96.8% for decisions and 99.4% for advice. Cases of disagreement were resolved through discussion. The final sample consisted of *N* = 301 participants, who were randomly assigned to the Increase IU condition (*n* = 101), Decrease IU condition (*n* = 101), or control condition (*n* = 99). Mean age was 31.3 years (*SD* = 10.9). The gender ratio was balanced (54.5% female, 43.9% male, 1.3% diverse, 0.3% not specified) and education levels were high, with 91.0% having a university degree or qualification to enter university.

### Measures and materials

#### Intolerance of uncertainty manipulation

To manipulate IU, we translated and adapted a method originally introduced by Britton and Davey (2014) to suit the German context at the time of data collection (October 2022). Participants read a fictitious report of a woman (Sarah) who described problems in her life, and were asked to generate advice for her. Then, they read five pieces of sample advice and were asked to revise their own advice if they wanted to. In the Increase IU condition, Sarah said that she was unable to react adequately to uncertain situations and behaved recklessly. Participants gave advice for reacting more strongly to uncertainty (i.e., being *more* intolerant towards uncertainty), and sample advice also pointed in this direction, for example: “You need to let yourself feel anxiety, stress and worry when you are confronted with uncertainty.” In the Decrease IU condition, in contrast, Sarah said that she was far too sensitive towards uncertainty and behaved overcautiously. Consequently, participants' advice should encourage Sarah to tolerate uncertainty better, which was also evident in the sample advice, for example: “Usually, what is uncertain at first turns out to be totally harmless.” (cf., Britton & Davey, 2014). A control condition, identical in structure but unrelated to uncertainty, was also implemented. In the control condition, the protagonist wanted to improve her eating habits.

To check the success of the manipulation, IU was measured using three items (Appel et al., 2024) adapted from the German translation (Gerlach et al., 2008) of the Intolerance of Uncertainty Scale (Freeston et al., 1994; e.g., “Uncertainty makes me uneasy, anxious, or stressed”). The following introduction served to capture situational IU (i.e., experienced in the moment): “To what extent do you agree with the following statements right now in this moment?” Participants used a slider bar ranging from 0 (*strongly disagree*) to 100 (*strongly agree*; Britton & Davey, 2014) to answer the items. Items were averaged and internal consistency was adequate, Cronbach's *α* = .77.

#### Decision task and indecisiveness measurement

To assess situational indecisiveness, participants were confronted with two decisions: First, they wrote about a personal important decision they were facing but had not made yet. Examples were given to prompt relevant descriptions (e.g., “Should I change jobs?”). Participants had to spend at least 30 s writing before being able to proceed. In the second decision task, participants chose between four lotteries, each offering different prize amounts and winning probabilities (4 ₤ at 40%, 5.30 ₤ at 30%, 8 ₤ at 20%, 16 ₤ at 10%). The expected value (prize × probability) was constant. Exploratorily, seven items were administered measuring perceptions of each decision (i.e., uncertainty, difficulty, and importance, e.g., “To me, the decision involves uncertainty”). Since the items were semantically related and had acceptable internal consistency within and across conditions (.70 ≤ Cronbach's *α* ≤ .76), they were averaged per decision to form two mean scores. Additionally, in order to ensure a minimum level of engagement with the description of their own decision, participants indicated to what extent they had thought vividly about the decision. All items were answered using a 1 (*strongly disagree*) to 5 (*strongly agree*) Likert‐type scale.

To assess situational indecisiveness, we presented a translated (Appel et al., 2024) adaptation of the shortened 11‐item version (Rassin et al., 2007) of the Indecisiveness Scale (Frost & Shows, 1993) after each decision task. Items were worded so that they referred to the respective decision at hand (e.g., “I become anxious when making *this* decision”, italics added; Appel & Gerlach, 2021). Participants answered the items on a Likert‐type scale from 1 (*strongly disagree*) to 5 (*strongly agree*). Items were summed to form a score. Cronbach's α was .85 for participants' own decision and .88 for the lottery decision.

### Procedure

After giving informed consent, participants answered demographic questions and then worked on the IU manipulation. They read Sarah's report, generated advice, read the sample advice and revised their advice if they wanted. Immediately afterwards, they indicated their state IU. Next, they described their personal upcoming decision and indicated their situational indecisiveness based on this decision. An attention check question was hidden among these items. Participants also answered the items measuring perceptions of the decision. Then, the lottery decision followed. After seeing the four options, but before making their choice, participants answered the same Indecisiveness Scale items, this time referring to the lottery decision. Only then did they take their decision and report their perceptions of the decision. To conclude, they answered questions capturing instruction comprehension and adherence (including whether they understood that the lottery decision was real). Participants were debriefed about the study's purpose and received a helpline contact in case the decision description had upset them.

### Data analysis

To test the success of the manipulation, we compared the score of the averaged situational IU items between conditions (Increase vs. Decrease vs. control) using a between‐subjects oneway‐ANOVA. For the main analyses, we used a between‐subjects oneway‐MANOVA with both indecisiveness scores as dependent variables to test differences between conditions (Increase vs. Decrease vs. control). The respective statistics for MANOVAs (Wilk's Lambda, Roy's Largest Root) were selected following the recommendations by Ateş et al. (2019). Planned contrasts compared both the Increase and Decrease condition with the control condition. Follow‐up tests other than planned contrasts were Holm‐Bonferroni‐corrected. Exploratory *t*‐tests comparing all conditions for each decision separately were one‐tailed due to our directional hypotheses. When Levene's test indicated statistically significant heterogeneity of variances, corrected *df*s, *t*‐ and *p*‐values were used. Additionally, we exploratorily conducted a mediation analysis for each decision, testing an indirect effect of the experimental manipulation on indecisiveness via situational IU. As the independent variable for the exploratory mediation, we created a dummy variable comparing the Increase condition (= 1) vs. Decrease condition (= 0) since the MANOVA revealed the largest indecisiveness differences between these conditions (see Results). Given that our design is experimental and participants were randomly assigned to conditions, we ensured the temporal sequence required for the mediation analysis (Maxwell et al., 2011). To assess the robustness of the results, we repeated analyses excluding multivariate outliers. We identified *n* = 12 multivariate outliers by determining each participant's Mahalanobis distance for all continuous variables. We then tested whether the Mahalanobis distance fell below a critical probability threshold (*p* < .001). For further robustness tests, we reran analyses excluding participants who did not believe the lottery decision was real (i.e., that money was actually paid), and including participants who did not describe a decision or could “not at all” identify with the description of their own decision, as had been preregistered. Unless otherwise noted, conclusions were not sensitive to these exclusions. More details on these robustness checks are given in Data S2. We carried out analyses in IBM SPSS V. 28, except the mediation analyses, which we conducted using PROCESS, V. 4.0, with the indirect effects calculated based on bootstrapping (Hayes, 2017; Model 4, 5000 bootstraps).

## RESULTS

Table 1 shows descriptive values for the dependent variables. The manipulation check indicated statistically significant differences in IU between conditions. Post‐hoc tests demonstrated higher IU in the Increase condition as compared to both the Decrease condition, *t*_*corrected*_(187.21) = 3.29, *p* < .002, *d* = 0.46, and the control condition, *t*_*corrected*_ (181.53) = 3.30, *p* = .001, *d* = 0.47, while the difference between the Decrease and control conditions was not statistically significant, *t*(198) = 0.06, *p* = .48.

**TABLE 1:** Means and Standard Deviations of dependent variables.

	Increase (*n* = 101)	Decrease (*n* = 101)	Control (*n* = 99)	Total sample (*N* = 301)
IU (0–100)	56.60 (18.30)	46.74 (23.91)	46.54 (24.45)	49.98 (22.80)
Indecisiveness‐own (11–55)	36.29 (8.34)	34.35 (8.67)	36.67 (7.36)	35.76 (8.18)
Indecisiveness‐lottery (11–55)	22.66 (7.79)	18.70 (6.95)	21.07 (7.42)	20.81 (7.55)

Abbreviation: IU, Intolerance of uncertainty, “right now in this moment”.

The main analysis showed a statistically significant difference between the conditions on the combined indecisiveness scores, *F*(2, 298) = 7.83, *p* < .001, partial η^2^ = .050, Roy's Largest Root = .053. Two subsequent MANOVAs on the combined indecisiveness scores served as planned contrasts between the control condition and both the Increase and Decrease condition. Contrary to H2, the difference in indecisiveness between the control and the Increase conditions was not statistically significant, *F*(2, 197) = 1.29, *p* = .277, partial η^2^ = .013, Wilk's Λ = .987. In line with H3, however, indecisiveness in the Decrease condition was statistically significantly lower than in the control condition, *F*(2, 197) = 3.80, *p* = .024, partial η^2^ = .037, Wilk's Λ = .963. Because the results did not support H2, H1 could not be automatically accepted. Therefore, an additional post‐hoc MANOVA compared the combined indecisiveness scores in the Increase vs. Decrease condition. The MANOVA showed a statistically significant difference, with higher scores in the Increase condition, *F*(2, 199) = 7.44, *p* < .001, partial η^2^ = .070, Wilk's Λ = .930, supporting H1. Exploratory post‐hoc *t*‐tests looking at each indecisiveness score separately comparing all conditions revealed that the effect found in the MANOVA was strongest for the lottery decision (Table 2).

**TABLE 2:** Post‐hoc comparisons of indecisiveness scores between conditions per decision.

Comparison	*df*	Own decision			Lottery decision		
		*t*	*p*	*d*	*t*	*p*	*d*
Increase v Decrease	200	1.62	.105	0.23	3.81	<.001	0.54
Increase v control	198	−0.34	.367	−0.05	1.48	.210	0.21
Decrease v control	198	−2.04	.084	−0.29	−2.33	.050	−0.33

*Note*: *p*‐values are one‐tailed and Bonferroni‐Holm corrected for multiple comparisons.

**FIGURE 1:**
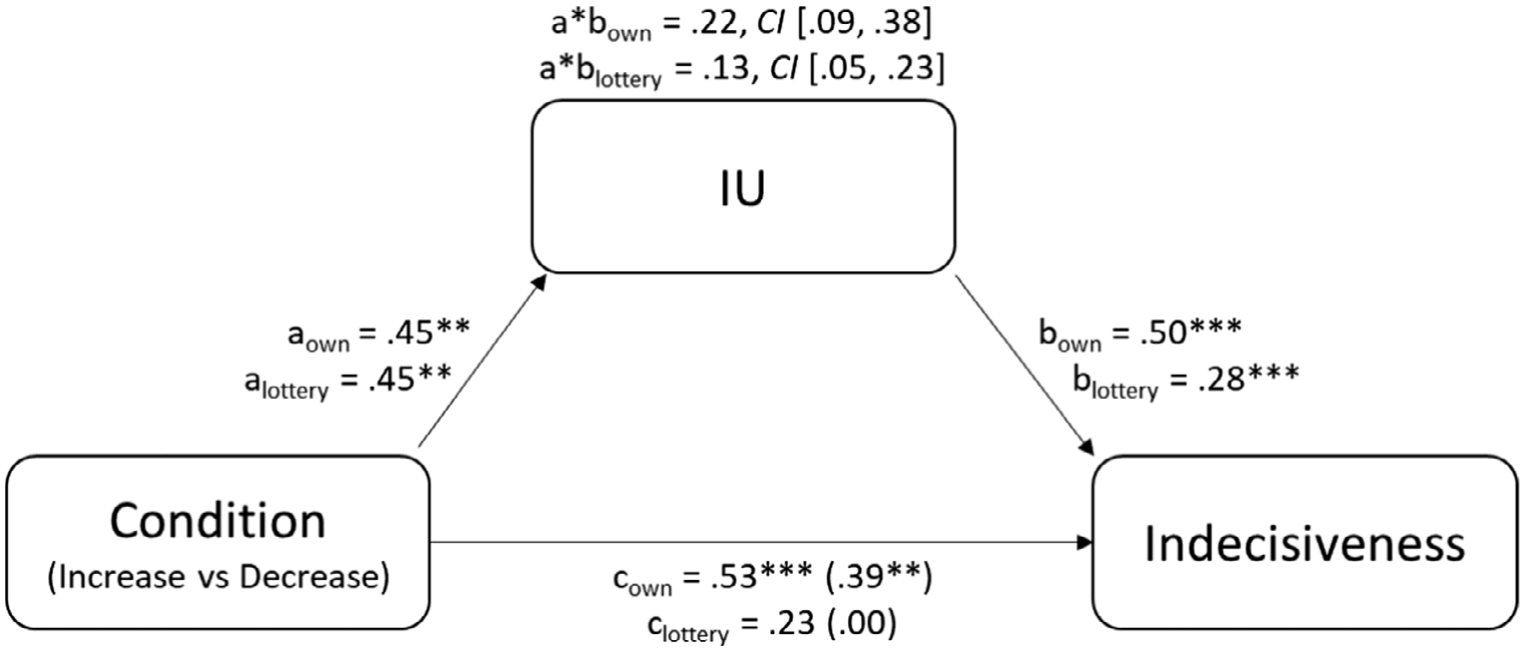
Illustration of mediation model for both decisions. Condition dummy coded (Increase condition = 1, Decrease condition = 0). **p < .01. ***p < .001.

Table 3 shows the results for the mediation analysis. In both decisions, there was an indirect effect of the condition (Increase vs. Decrease condition) on indecisiveness via IU. This indirect effect emerged in both analyses although the main effect of the manipulation was not statistically significant for participants' own decision (cf. Table 2). For participants' own decision, the direct effect was reduced in size compared to the total effect, but since the total effect was not statistically significant, this change cannot be interpreted. For the lottery decision, the direct effect was also reduced in size compared to the total effect, but remained statistically significant, indicating partial mediation. Figure 1 illustrates the mediation.

**TABLE 3:** Mediation model for each decision comparing the Increase vs. Decrease condition.

	Own decision				Lottery decision			
	*B*	*SE*	*β*	*CI (LL, UL)*	*B*	*SE*	*β*	*CI (LL, UL)*
DV: IU								
Condition (a‐path)	9.86	3.00	.45	3.95, 15.77	9.86	3.00	.45	3.95, 15.77
DV: Indecisiveness								
IU^a^	0.20	0.02	.50	0.15, 0.24	0.11	0.02	.33	0.07, 0.16
DV: Indecisiveness (mediation model)								
IV: Conditon (direct effect)	0.02	1.08	.00	−2.10, 2.14	2.99	1.03	.39	0.97, 5.02
M: IU (b‐path)	0.19	0.02	.50	0.15, 0.24	0.10	0.02	.28	0.05, 0.14
Indirect effect	1.92	0.63	.22	0.76, 3.24	0.97	0.37	.13	0.35, 1.78
Total effect	1.94	1.20	.23	−0.42, 4.30	3.96	1.04	.52	1.91, 6.01

*Note*: Condition dummy coded (Increase condition = 1, Decrease condition = 0).

Abbreviations: CI, 95% confidence intervals (referring to unstandardized coefficients); DV, dependent variable; IV, independent variable; M, mediator.

^a^ Not controlling for IU.

Sensitivity analyses (details in Data S2) to check the robustness of the findings were completely consistent with the results from the main analysis, with one minor exception: When excluding multivariate outliers, the difference in indecisiveness scores between the Increase and control condition was statistically significant, *F*(1, 188) = 3.25, *p* = .041, partial η^2^ = .033, Wilk's Λ = .967, with higher scores in the Increase condition. The results aligned with H2, but contradicted the main analysis. Conversely, the difference between the control and Decrease condition was not statistically significant, *F*(2, 188) = 2.35, *p* = .098, partial η^2^ = .024, Wilk's Λ = .976, which also deviated from the main analysis. Importantly, the crucial comparison between the Increase and Decrease condition revealed the same statistically significant difference as predicted in H1 and found in the main analyses, with higher indecisiveness scores in the Increase condition, *F*(2, 193) = 6.77, *p* = .001, partial η^2^ = .066, Wilk's Λ = .934.

An exploratory 2 × 3 ANOVA with type of decision (own vs. lottery) as a within‐subjects factor and condition (Increase vs. Decease vs. control) as a between‐subjects factor was conducted on the mean scores of the seven items measuring perceptions of both decisions. The results of the ANOVA indicated more uncertainty, difficulty, and importance for participants' own compared to the lottery decision, *F*(1, 298) = 1839.15, *p* < .001, partial η^2^ = .861. There was no effect of experimental condition, *F*(2, 298) = 2.73, *p* = .067, partial η^2^ = .018, and no interaction between both factors, *F*(2, 298) = 1.24, *p* = .292, partial η^2^ = .008.

## DISCUSSION

In this study, we used an experimental manipulation of IU to assess its causal effect on indecisiveness in two personally relevant decisions. According to the manipulation check, the manipulation indeed affected IU: situational IU after the manipulation was higher in the Increase condition than in both the Decrease and control conditions. The latter two did not show a statistically significant difference.

Most importantly, increased IU led to more indecisiveness compared to decreased IU, showing a causal effect of IU on indecisiveness. More specifically, the increase in indecisiveness in the Increase compared to the Decrease condition was statistically significant for the lottery decision, but not for the personal decision. The effect size for the Increase vs. Decrease comparison in the lottery decision was *d* = 0.54, indicating a moderate effect. Although this difference was not statistically significant for the personal decision, it was still numerically positive (*d* = 0.23). The two decisions may differ in sources of error variance, explaining the absence of a statistically significant effect in the personal decision. The lottery decision provided the same four choices to everyone. It also ensured personal relevance as it directly affected participants' potential winnings. In contrast, the personal decisions varied widely (e.g., “Should I move in together with my girlfriend?”; “Should I look for a new GP?”, “Should I sell my car?”). This wider range may have created more error variance, making it more challenging to detect the impact of the IU manipulation. The larger standard deviation in indecisiveness scores for personal decisions supports this reasoning.

Surprisingly, indecisiveness levels in the control condition were similar to the Increase condition. Although indecisiveness in the control condition was not as high as in the Increase condition, this pattern was unexpected.

To further investigate the impact of IU on indecisiveness, we conducted an exploratory mediation analysis. The mediation analysis revealed that the differences in indecisiveness between the Increase and Decrease conditions were partially mediated by IU. This finding suggests two important insights: First, it supports the assumed mechanism of the manipulation, showing that, in fact, IU explained the observed changes in indecisiveness. Second, it showed that, whereas the effect of the manipulation was not statistically significant for the personal decision in the main analysis, indecisiveness was nonetheless mediated by IU. However, given the somewhat unclear result pattern regarding the control condition, and because the mediation analysis was not preregistered, future studies should strive to replicate this finding.

Overall, most effect sizes were small, with only the contrast in indecisiveness scores between the Increase and Decrease conditions being medium‐sized. Given our realistic measurement of indecisiveness (related to actual decisions), the rather small effect sizes are not surprising. They reflect the complex determinacy of psychological processes (Funder & Ozer, 2019), wherein IU is most likely one of several factors influencing indecisiveness (van van Randenborgh et al., 2010). This reasoning is also in line with Rassin's (2007) theory of indecisiveness, which specifies other antecedents of indecisiveness in addition to IU, such as the disposition to always excessively look for the ultimate best choice option (“maximizing”, Schwartz et al., 2002).

The study incorporates particularly strong methodology. Most importantly, it allows testing the causal effect of IU on indecisiveness by employing an experimental manipulation of IU (Britton & Davey, 2014). Also, the use of real decisions strengthens ecological validity. Thanks to a relatively large sample size, statistical power was adequate, making the results more reliable. Rigorous data quality checks further contributed to the reliability, and sensitivity analyses indicated that the main findings were robust to excluding unusual cases. However, some limitations must also be considered. First, we did not use a pre‐selected sample with high IU levels or mental disorders associated with elevated IU. A pre‐selected sample would have allowed investigating whether the results extend to those who are particularly burdened with high IU and indecisiveness. On the other hand, studying an unselected sample is valuable because it provides insights into the general prevalence of the observed effects. Another drawback of the present study sample is the relatively narrow age range and the very high education level, making generalization to the general population questionable. The sample is, however, less homogeneous and therefore somewhat more representative of the general population compared to student samples (Henrich et al., 2010). Future replication studies should be conducted with more representative samples and samples with higher IU levels. Another limitation could be the IU manipulation. Being very specific and referring to a clearly fictitious case report, the manipulation cannot be expected to have a long‐lasting effect. Investigating how profound interventions against IU (e.g., Mofrad et al., 2020) affect indecisiveness would therefore be an important addition.

It is important to highlight that this study provides initial evidence on the causal role of IU in indecisiveness. However, the exact extent and possible boundary conditions of this effect warrant further investigation to strengthen the evidence. For example, we specifically targeted IU by implementing an established IU manipulation and conducting the respective manipulation check. Future research could incorporate related but distinct constructs, such as anxiety‐related measures, alongside the included variables (Koerner et al., 2017), to ensure that these do not account for the effect. Examinations of specificity are also important for determining the role of IU within the broader framework of higher‐order factors that contribute to mental health symptoms or maladaptive behaviours (Morriss, 2023). Furthermore, we refrained from measuring IU at the trait level to avoid overcomplicating the study design. Given the large sample size and the random assignment of participants to experimental conditions, substantial differences in trait IU levels between experimental groups are unlikely, but cannot be entirely ruled out. Also, this precluded the examination of potential interactions between state and trait levels of IU, despite evidence from previous studies indicating a relationship between the two (Appel et al., 2024). Future research could address this limitation by incorporating trait IU measures.

This being said, the results have important implications both theoretically and practically. On a theoretical level, the present data support the idea that IU is a causal predisposing risk factor for indecisiveness (Rassin, 2007). This insight is furthermore in line with IU being an important transdiagnostic risk factor for psychopathology. Conversely, IU is also amenable to therapeutic interventions and can be effectively modified, even in cases of severe manifestations, such as in GAD (Wilson et al., 2023), and, more importantly, IU reductions explain variance in the improvement of various disorders and symptoms (Miller & McGuire, 2023).

The present findings, in turn, demonstrate that IU specifically is a driver for indecisiveness. Indecisiveness in problematic forms or at excessive levels is an important symptom because it is also found transdiagnostically and because impaired decision making often has severe consequences (e.g., Germeijs & De Boeck, 2002). For example, putting off important decisions could aggravate distress for a person already suffering from a mental disorder, potentially constituting a maintaining factor (Ferrari, 1994). Thus, the study's findings contribute to the growing evidence supporting IU reduction as a valuable treatment target. If IU can be therapeutically modified, and IU causally influences indecisiveness, it is likely that excessive indecisiveness also responds to IU‐targeted treatments. The relevance of IU also applies to interventions against problematic indecisiveness in the non‐pathological domain, especially where uncertainty is sometimes unavoidable, e.g., in medical decision‐making. Here, too, approaches to reduce IU have been proposed (Gheihman et al., 2020). According to the present study, these can help improve problems with indecisiveness.

## CONCLUSIONS

This study causally demonstrates that an experimental IU manipulation affects indecisiveness. Exploratory evidence from mediation analysis suggests that situational IU indeed serves as a mediator in this effect. The outcomes suggest that enhancing tolerance for uncertainty is a promising strategy for reducing indecisiveness. Since indecisiveness can be a substantial burden both generally and within various mental disorders, these results highlight the importance of interventions targeting IU across different diagnoses. Future research should aim to replicate these findings using samples selected for high levels of IU and indecisiveness and implement more robust interventions designed to effectively alter IU.

## AUTHOR CONTRIBUTIONS

**Helmut Appel:** Conceptualization; methodology; software; data curation; formal analysis; project administration; writing – original draft; investigation. **Alexander L. Gerlach:** Conceptualization; funding acquisition; writing – review and editing; supervision.

## CONFLICT OF INTEREST STATEMENT

The authors declare that they have no conflict of interest.

## Supporting information


Data S1.

Data S2.

## Data Availability

The data that support the findings of this study are available from the corresponding author upon reasonable request.
