# Supplementary material for: Intolerance of uncertainty causally affects indecisiveness
Source: Br J Clin Psychol. 2025 Mar 11;64(3):806–16. doi: 10.1111/bjc.12534 (PMC12334985; doi:10.1111/bjc.12534)
Supplement: Supplementary file 1 — Data S1. [file BJC-64-806-s001.pdf]

Electronic Supplemental Materials

ESM 1 – Deviations from Preregistration

Article title: Intolerance of Uncertainty Causally Affects Indecisiveness

Author names: Appel, Helmut and Gerlach, Alexander L.

Affiliation and e-mail address of the corresponding author: Institute of Clinical Psychology and Psychotherapy, University of Cologne, Germany; helmut.appel@uni-koeln.de

Introduction:

In this document, we transparently report all deviations from the preregistration of this study. The structure is adapted from:

Claesen, A., Gomes, S., Tuerlinckx, F., & Vanpaemel, W. (2021). Comparing dream to reality: an assessment of adherence of the first generation of preregistered studies. *Royal Society Open Science, 8*(10), 211037.

Harris, Campbell, Flake, Fried, Beck, and Kline at the APS Hackathon 2019: Best Research Practices Made Easy (for more details see: https://osf.io/c8xbp/).

Overview

Components	Were there deviations? (no, minor, major)	If yes - describe the deviation(s) or link to location in manuscript/supplementary	Rationale for deviation or link to location in manuscript/supplementary	How might the results be different if you had not deviated
Hypothesis / research question	minor	We conducted additional analyses not foreseen in the preregistration (mediation analysis)	These analyses examined interesting additional aspects which we had anticipated at the stage of planning and preregistration	We labeled all conclusions derived from these analyses as preliminary and mentioned the need to replicate them in future studies
Variables	no			
Direction of effect	no			
Operationalization	no			
Sample size	no			
Exclusion criteria	minor	According to preregistration, the following exclusion criteria were originally only to be applied in analyses involving indecisiveness	Clarity and consistency between analyses	We tested any effects in a robustness test / sensitivity analysis and found that results were not affected (see ESM 2 – Additional Analyses)

		referring to participants' own decision, but for simplicity, we decided to completely exclude these cases:<br><br>- indicated they could "not at all" (1 on a scale from 1-5) think vividly about the decision<br><br>- did not describe a decision		
Procedure	no			
Statistical model	no			
