# Supplementary material for: Intolerance of uncertainty causally affects indecisiveness
Source: Br J Clin Psychol. 2025 Mar 11;64(3):806–16. doi: 10.1111/bjc.12534 (PMC12334985; doi:10.1111/bjc.12534)
Supplement: Supplementary file 2 — Data S2. [file BJC-64-806-s002.pdf]

Electronic Supplemental Materials

ESM 2 –Additional Analysis (Robustness Checks)

Article title: Intolerance of Uncertainty Causally Affects Indecisiveness

Author names: Appel, Helmut and Gerlach, Alexander L.

Affiliation and e-mail address of the corresponding author: Institute of Clinical Psychology and Psychotherapy, University of Cologne, Germany; helmut.appel@uni-koeln.de

Note

First, in line with preregistration, we identified multivariate outliers by determining each participant's Mahalanobis distance for all continuous variables and verifying whether it fell below a critical probability threshold (< .001). To assess the robustness of the results, we repeated analyses without multivariate outliers. We identified n = 12 multivariate outliers.

Next, in line with preregistration, we excluded n = 44 participants who did not believe the lottery was real (i.e., money was actually being paid) and repeated analyses.

Also, we repeated analyses including participants who had indicated that they could "not at all" imagine their decision vividly or did not describe a valid decision. In the main analysis, we excluded these participants for the sake of simplicity. However, because we had preregistered that we would only exclude these cases from analyses involving indecisiveness scores referring to participants' own decision description, we repeated analyses including these n = 10 cases.

Results of Robustness Check / Sensitivity Analysis Excluding Multivariate Outliers

The analysis showed a statistically significant difference between the conditions on the combined indecisiveness scores, $F(2, 286) = 7.40$, $p < .001$, partial $\eta^2 = .049$, Roy's Largest Root = .052. Two subsequent MANOVAs on the combined indecisiveness scores served as planned contrasts between the control condition and both the Increase and Decrease condition. In contrast to the main analysis, but in line with H2, the difference between the control and the Increase condition was

significant, $F(2, 188) = 3.25$, $p = .041$, partial $\eta^2 = .033$, Wilk's $\Lambda = .967$. On the other hand, in contrast with the main analysis and contradicting H3, indecisiveness was only marginally significantly higher in the control compared to the Decrease condition, $F(2, 188) = 2.35$, $p = .098$, partial $\eta^2 = .024$, Wilk's $\Lambda = .976$. Because the results were not in line with H3, H1 could not be automatically accepted, so an additional post-hoc MANOVA compared the combined indecisiveness scores in the Increase vs Decrease condition. It showed significantly higher scores in the Increase condition, $F(2, 193) = 6.77$, $p = .001$, partial $\eta^2 = .066$, Wilk's $\Lambda = .934$, supporting H1. Exploratory post-hoc *t*-tests looking at each indecisiveness score separately comparing all conditions revealed that the effect found in the MANOVA was strongest for the lottery decision (Table ESM 2-1).

Table ESM 2-1

Post-hoc Comparisons of Indecisiveness Scores Between Conditions per Decision

Decision	Comparison	*t*	*df*	*p*
own	Increase v Decrease	1.36	194	.174
	Increase v control	-0.54	189	.297
	Decrease v control	-1.96	189	.100
lottery	Increase v Decrease	3.67	194	<.001
	Increase v control	2.32	189	.055
	Decrease v control	-1.47	189	.216

Note. *p*-values one-tailed, Bonferroni-Holm corrected for multiple comparisons.

Table ESM 2-2 shows the results for the mediation analysis. In both decisions, there was an indirect effect of the condition (Increase vs Decrease condition) on indecisiveness via IU. This was true although the main effect of the manipulation was not significant for participants' own decision (cf. Table ESM 2-1). For participants' own decision, the direct effect was reduced in size compared to the total effect, but since the total effect was not significant, this change cannot be interpreted. For the lottery decision, the direct effect was also reduced in size compared to the total effect, but remained significant, indicating partial mediation.

Table ESM 2-2

Mediation Model for Each Decision Comparing the Increase vs Decrease Condition

	Own decision				Lottery decision			
	B	*SE*	β	*CI (LL, UL)*	*B*	*SE*	β	*CI (LL, UL)*
DV: IU								
Condition (a-path)	9.54	3.06	.44	3.50, 15.58	9.54	3.06	.44	3.50, 15.58
DV: Indecisiveness								
IU[a]	0.19	0.02	.50	0.14, 0.24	0.12	0.02	.34	0.07, 0.17
DV: Indecisiveness (mediation model)								
IV: Conditon (direct effect)	-0.20	1.08	-.02	-2.33, 1.94	2.91	1.04	.38	0.86, 5.00
M: IU (b-path)	0.19	0.02	.50	0.14, 0.24	0.10	0.02	.30	0.06, 0.15
Indirect effect	1.84	0.64	.22	0.65, 3.13	0.99	0.39	.13	0.31, 1.85
Total effect	1.64	1.21	.19	-0.74, 4.02	3.90	1.06	.51	1.80, 5.99

Note. DV = dependent variable, IV = independent variable, M = mediator, CI = 95% confidence intervals (referring to unstandardized coefficients).

[a]not controlling for IU

Results of Robustness Check / Sensitivity Analysis Excluding Participants who Did Not Believe the Lottery was Real

The analysis showed a statistically significant difference between the conditions on the combined indecisiveness scores, $F(2, 254) = 7.33$, $p < .001$, partial $\eta^2 = .055$, Roy's Largest Root = .058. Two subsequent MANOVAs on the combined indecisiveness scores served as planned contrasts between the control condition and both the Increase and Decrease condition. In contrast to H2, the difference between the control and the Increase condition was not significant, $F(2, 168) = 1.41$, $p = .247$, partial $\eta^2 = .017$, Wilk's $\Lambda = .983$. On the other hand, in line with H3, indecisiveness was significantly higher in the control compared to the Decrease condition, $F(2, 169) = 3.18$, $p = .044$, partial $\eta^2 = .036$, Wilk's $\Lambda = .964$,. Because the results were not in line with H2, H1 could not be automatically accepted, so an additional post-hoc MANOVA compared the combined

indecisiveness scores in the Increase vs Decrease condition. It showed significantly higher scores in the Increase condition, $F(2, 168) = 7.04$, $p = .001$, partial $\eta^2 = .077$, Wilk's $\Lambda = .923$, supporting H1. Exploratory post-hoc *t*-tests looking at each indecisiveness score separately comparing all conditions revealed that the effect found in the MANOVA was strongest for the lottery decision (Table ESM 2-3).

Table ESM 2-3

Post-hoc Comparisons per Decision

**Decision**	**Comparison**	***t***	***df***	***p***
own	Increase v Decrease	1.17	169	.242
	Increase v control	-0.38	169	.351
	Decrease v control	-1.6	170	.224
lottery	Increase v Decrease	3.76	169	< .001
	Increase v control	1.54	169	.189
	Decrease v control	-2.28	170	.060

Note. *p*-values one-tailed, Bonferroni-Holm corrected for multiple comparisons.

Table ESM 2-4 shows the results for the mediation analysis. In both decisions, there was an indirect effect of the condition (Increase vs Decrease condition) on indecisiveness via IU. This was true although the main effect of the manipulation was not significant for participants' own decision (cf. Table 2). For participants' own decision, the direct effect was reduced in size compared to the total effect, but since the total effect was not significant, this change cannot be interpreted. For the lottery decision, the direct effect was also reduced in size compared to the total effect, but remained significant, indicating partial mediation.

Table ESM 2-4

Mediation Model for Each Decision Comparing the Increase vs Decrease Condition

	Own decision				Lottery decision			
	B	*SE*	β	*CI (LL, UL)*	*B*	*SE*	β	*CI (LL, UL)*
DV: IU								
Condition (a-path)	9.99	3.23	.46	3.61, 16.36	9.99	3.23	.46	3.61, 16.36
DV: Indecisiveness								
IU[a]	0.20	0.03	.51	0.15, 0.25	0.11	0.03	.31	0.06, 0.16
DV: Indecisiveness (mediation model)								
IV: Conditon (direct effect)	-0.50	1.17	-.06	-2.80, 1.80	3.24	1.09	.43	1.08, 5.40
M: IU (b-path)	0.20	0.03	.51	0.15, 0.26	0.09	0.03	.26	0.04, 0,14
Indirect effect	2.04	0.71	.24	0.74, 3.50	0.89	0.36	.12	0.27, 1.67
Total effect	1.54	1.31	.18	-1.05, 4.12	4.13	1.10	.55	1.96, 6.30

Note. DV = dependent variable, IV = independent variable, M = mediator, CI = 95% confidence intervals (referring to unstandardized coefficients).

[a]not controlling for IU

Results of Robustness Check / Sensitivity Analysis Including "Low Vividness" and No Valid Decision Description Cases

The analysis showed a statistically significant difference between the conditions on the combined indecisiveness scores, $F(2, 308) = 8.09$, $p < .001$, partial $\eta^2 = .050$, Roy's Largest Root = .052. Two subsequent MANOVAs on the combined indecisiveness scores served as planned contrasts between the control condition and both the Increase and Decrease condition. In contrast to H2, the difference between the control and the Increase condition was not significant, $F(2, 203) = 1.23$, $p = .295$, partial $\eta^2 = .012$, Wilk's $\Lambda = .967$ On the other hand, in line with H3, indecisiveness was significantly higher in the control compared to the Decrease condition, $F(2, 194) = 3.29$, $p = .039$, partial $\eta^2 = .033$, Wilk's $\Lambda = .967$,. Because the results were not in line with H2,

H1 could not be automatically accepted, so an additional post-hoc MANOVA compared the combined indecisiveness scores in the Increase vs Decrease condition. It showed significantly higher scores in the Increase condition, $F(2, 199) = 7.06$, $p = .001$, partial $\eta^2 = .066$, Wilk's $\Lambda = .934$, supporting H1. Exploratory post-hoc *t*-tests looking at each indecisiveness score separately comparing all conditions revealed that the effect found in the MANOVA was strongest for the lottery decision (Table ESM 2-5).

Table ESM 2-5

Post-hoc Comparisons per Decision

Decision	Comparison	*t*	*df*	*p*
own	Increase v Decrease	1.48	208	.210
	Increase v control	-0.68	204	.250
	Decrease v control	-2.21	204	.056
lottery	Increase v Decrease	3.85	208	<.001
	Increase v control	1.26	204	.210
	Decrease v control	-2.59	204	.025

Note. *p*-values one-tailed, Bonferroni-Holm corrected for multiple comparisons.

Table ESM 2-6 shows the results for the mediation analysis. In both decisions, there was an indirect effect of the condition (Increase vs Decrease condition) on indecisiveness via IU. This was true although the main effect of the manipulation was not significant for participants' own decision (cf. Table 2). For participants' own decision, the direct effect was reduced in size compared to the total effect, but since the total effect was not significant, this change cannot be interpreted. For the lottery decision, the direct effect was also reduced in size compared to the total effect, but remained significant, indicating partial mediation.

Table ESM 2-6

Mediation Model for Each Decision Comparing the Increase vs Decrease Condition

	Own decision				Lottery decision			
	B	***SE***	**β**	***CI (LL, UL)***	***B***	***SE***	**β**	***CI (LL, UL)***
DV: IU								
Condition (a-path)	10.10	2.97	.46	4.24, 15.95	10.10	2.97	.46	4.24, 15.95
DV: Indecisiveness								
IU[a]	0.19	0.02	.50	0.15, 0.24	0.12	0.02	.34	0.07, 0.16
DV: Indecisiveness (mediation model)								
IV: Conditon (direct effect)	-0.23	1.05	-.03	-2.29, 1.84	2.89	1.00	.38	0.91, 4.86
M: IU (b-path)	0.19	0.02	.50	0.15, 0.24	0.10	0.02	.30	0.06, 0.15
Indirect effect	1.96	0.62	.23	0.80, 3.25	1.04	0.38	.14	0.39, 1.87
Total effect	1.73	1.17	.20	-0.57, 4.04	3.92	1.02	.51	1.91, 5.93

Note. DV = dependent variable, IV = independent variable, M = mediator, CI = 95% confidence intervals (referring to unstandardized coefficients).

[a]not controlling for IU
